# Exploring a genetic basis for the metabolic perturbations in ME/CFS using UK biobank

**DOI:** 10.1016/j.isci.2025.114316

**Published:** 2025-12-03

**Authors:** Katherine Huang, Muhammad Muneeb, Natalie Thomas, Elena K. Schneider-Futschik, Paul R. Gooley, David B. Ascher, Christopher W. Armstrong

**Affiliations:** 1 Department of Biochemistry and Pharmacology, Bio21 Molecular Science and Biotechnology Institute, University of Melbourne, Parkville, VIC 3052, Australia; 2 School of Chemistry and Molecular Biosciences, University of Queensland, Brisbane City, QLD 4072, Australia; 3 Computational Biology and Clinical Informatics, Baker Heart and Diabetes Institute, Melbourne, VIC 3004, Australia; 4 Baker Department of Cardiometabolic Health, University of Melbourne, Parkville, VIC 3010, Australia

**Keywords:** health sciences, medicine, genetics

## Abstract

Myalgic encephalomyelitis/chronic fatigue syndrome (ME/CFS) is a clinically heterogeneous disease lacking approved therapies. To assess genetic susceptibility toward a specific metabolic phenotype, we performed a genome-wide association study on plasma biomarker levels (mGWAS) in patients with ME/CFS (*n* = 875) and healthy controls (HCs) (*n* = 36,033). We identified 112 significant SNP-biomarker associations in ME/CFS, compared with 4,114 in HCs. Two SNPs specific to ME/CFS, mapping to *HSD11B1* and *SCGN*, were associated with phospholipids in extra-large very low-density lipoproteins (VLDLs) and total fatty acids, respectively. Genetic effects of VLDL associations were among the least correlated between ME/CFS and HCs. Heterogeneity tests found differential effects for several lipid traits at *ADAP1*, *NR1H3,* and *CD40*, which are involved in immune regulation. ME/CFS mGWAS summary statistics were decomposed to uncover shared genetic-metabolic patterns, where enrichment analysis highlighted pathways in lipid metabolism, neurotransmitter transport, and inflammation. These findings provide a genetic and molecular rationale for patient heterogeneity and suggest a polygenic predisposition in which many small-effect variants may jointly perturb metabolic mechanisms.

## Highlights

• ME/CFS shows distinct genetic influences on metabolic regulation
• Lipid and hormone-related pathways emerge as key areas of interest
• Many small genetic effects may collectively disrupt metabolic resilience in ME/CFS

## Graphical abstract

**Figure:**
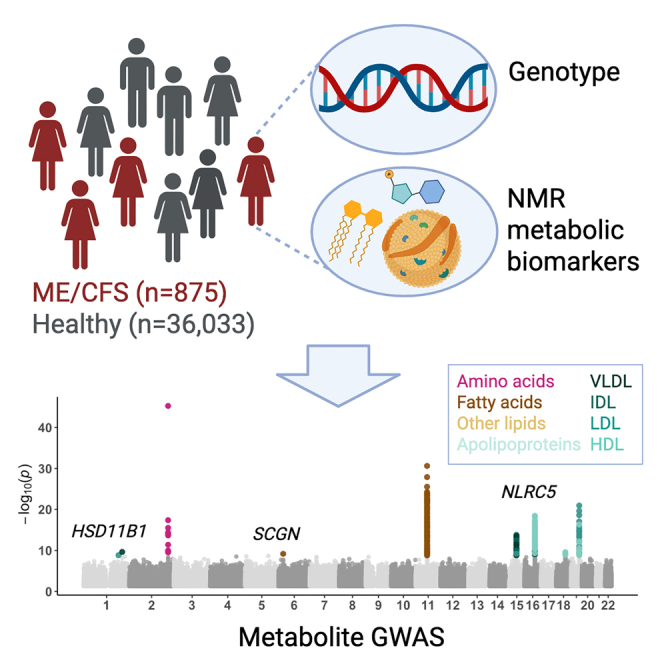

## Introduction

Myalgic Encephalomyelitis/Chronic Fatigue Syndrome (ME/CFS) is a complex and debilitating illness, characterized by idiopathic fatigue, post-exertional malaise, and unrefreshing sleep. ME/CFS is triggered by viral/bacterial infection, trauma, or toxin exposure, setting off a cascade of dysfunctional biological processes that manifest into additional widespread symptoms, including brain fog, muscle weakness, gastrointestinal issues, and orthostatic intolerance.^1^ While a definitive understanding of ME/CFS pathophysiology remains elusive, numerous hypotheses have been proposed with multi-omics technologies.^2^^,^^3^

The static genome and the dynamic metabolome, which amalgamate the influences of genetics and external environmental factors, offer insights into an individual’s health. In ME/CFS, metabolomic and transcriptomic studies have identified disruptions in energy metabolism, including impaired glycolysis,^4^ inefficient TCA cycle processes,^5^^,^^6^ altered lipid metabolism,^7^^,^^8^ dysregulated urea cycle,^5^^,^^9^ immune system,^10^ inflammatory responses,^8^^,^^11^ and neuroendocrinology pathways,^12^ highlighting that biomolecular disturbances are central to the disease.

ME/CFS has a heritable component, as suggested by familial clustering and twin studies.^13^ Until recently, case-control genome-wide association studies (GWASs) had not identified single nucleotide polymorphisms (SNPs) reaching genome-wide significance, largely due to small sample sizes.^14^^,^^15^^,^^16^ These limitations have mostly been overcome by the DecodeME study (*n* = 15,579), which discovered eight loci associated with ME/CFS.^17^ This progress not only provides compelling evidence of a genetic predisposition, but also opens new opportunities to apply complementary approaches to explore potential genetic contributions that underlie inefficient metabolism mechanisms that may operate in ME/CFS.

Metabolite GWAS (mGWAS) in both healthy and diseased cohorts have identified hundreds of gene loci that influence circulating metabolite levels, which are involved in maintaining homeostasis or driving disease processes.^18^^,^^19^^,^^20^^,^^21^^,^^22^ Unlike GWAS for diseases, mGWAS often detects larger effect sizes due to the direct connection between metabolite levels and the biological pathways affected by genetic variation.^23^ In addition, by treating metabolite levels as quantitative traits, mGWAS can uncover SNPs that define metabolic subgroups within a heterogeneous condition such as ME/CFS. This stratified approach enables the identification of genetic variants that may only be present in a proportion of patients with ME/CFS, offering a more precise understanding of disease mechanisms, subgroups, and potential targets for personalized interventions.

Using the UK Biobank (UKB), we investigated the associations between metabolic biomarker levels measured through nuclear magnetic resonance (NMR) spectroscopy of plasma samples and genotype data in both ME/CFS and healthy controls (HCs). Specifically, we (1) identified unique and overlapping SNP-biomarker associations in both groups and assigned genes based on proximity and functional relevance, (2) highlighted the potential downstream consequences of different marginal effects for the genetic associations, and (3) applied a hypothesis-free decomposition technique^24^ to elucidate biological pathways from summary statistics. Overall, this work exemplifies how the accumulation of subtle genetic disruptions across interconnected metabolic and signaling pathways may contribute to ME/CFS pathophysiology.

## Results

### Study population

Our study population was selected from the UKB, which is a large biomedical database with half a million participants, aged 39–70 years, comprising genetic, lifestyle, and electronic health records. The initial cohort included participants with baseline NMR metabolic biomarkers (*n* = 274,353), where 875 ME/CFS cases (median age of 56 years, 76% females) and 36,033 HCs (median age of 54 years, 53% females) that passed genetic quality control (See STAR Methods) were used as our final groups (Table 1). ME/CFS cases were identified from self-reported clinical diagnosis, which may include some misdiagnosed individuals. In addition, the UKB’s inherent volunteer bias toward healthier participants likely reflects an ME/CFS cohort with milder disease severity.

**Table 1:** Study population characteristics

	ME/CFS	Healthy controls	*p*-value
Sample size (n)	875	36033	–
Female (%)	75.2	53.2	1.06 × 10^−37^
Age at recruitment: Median (IQR)	56 (50–62)	54 (47–61)	4.31 × 10^−11^
Fasting time (hours): Median (IQR)	3 (2–4)	3 (2–4)	3.64 × 10^−2^
Disease duration (years): Median (IQR)	11.8 (6–18.2)	–	–
Physical measures (Median, IQR)			
Body mass index (kg/m^2^)	26.7 (23.9–30.2)	25.7 (23.5–28.5)	5.80 × 10^−11^
Diastolic blood pressure (mmHg)	80.5 (74–87.5)	80.5 (74.5–87.5)	0.737
Systolic blood pressure (mmHg)	133 (121–144.5)	133 (122.5–145.5)	0.113
Pulse rate (bpm)	71.5 (65–79.5)	67.5 (61–74.5)	3.68 × 10^−31^
Hand grip strength, left (kg)	24 (18–30)	30 (22–40)	1.33 × 19^−66^
Hand grip strength, right (kg)	26 (20–32)	32 (26–42)	6.42 × 10^−69^
Basal metabolic rate (kJ)	5845 (5326–6896)	6318 (5464–7623)	3.20 × 10^−17^
Lifestyle (%)^a^			
Alcohol drinker status			
Current	82.9	95.8	1.90 × 10^−95^
Never	5.6	2.3	
Previous	11.3	1.8	
Smoking status			
Current	8.8	10.8	0.102
Never	62.4	59.5	
Previous	28.6	29.4	
International physical activity questionnaire activity group^25^			
Low	25.4	12.9	1.33 × 10^−35^
Moderate	32.8	33.1	
High	21.6	37.1	
Pathology (Median, IQR)			
Alanine aminotransferase (U/L)	19.7 (15.1–26.8)	19.1 (14.7–25.7)	0.038
Alkaline phosphatase (U/L)	81.6 (67.4–99.4)	77.5 (65–91.9)	1.53 × 10^−8^
Aspartate aminotransferase (U/L)	24 (20.5–28)	23.8 (20.5–27.9)	0.425
C-reactive protein (mg/L)	1.4 (0.6–2.9)	1 (0.5–2.1)	3.37 × 10^−11^
Gamma glutamyltransferase (U/L)	23.4 (17.2–38)	23.4 (17.1–35.6)	0.357
Sex hormone binding globulin (nmol/L)	50.1 (34.6–71.2)	47.4 (33.9–66.8)	0.032
Testosterone (nmol/L)	1.3 (0.8–7.5)	3.3 (1–12.2)	1.32 × 10^−29^
Total bilirubin (μmol/L)	7.4 (5.9–9.6)	8.2 (6.6–10.5)	5.16 × 10^−14^
Urate (μmol/L)	277.9 (230.2–333)	292.4 (242–348.2)	7.36 × 10^−7^
Urea (mmol/L)	5 (4.3–5.8)	5.2 (4.5–6)	4.05 × 10^−4^
Vitamin D (nmol/L)	47.1 (31.8–62.5)	48.8 (34.5–63.7)	0.013
Leukocyte count (109 cells/L)	6.6 (5.6–7.8)	6.4 (5.5–7.6)	0.005
Lymphocyte count (109 cells/L)	1.9 (1.5–2.3)	1.8 (1.5–2.2)	0.038
Monocyte count (109 cells/L)	0.4 (0.3–0.5)	0.4 (0.3–0.5)	0.934
Neutrophil count (109 cells/L)	4 (3.3–4.9)	3.9 (3.2–4.8)	0.011
Comorbid conditions (%)			
Hypertension	25.5	–	–
Asthma	16.3	–	–
Depression	15.7	–	–
Osteoarthritis	13.3	–	–
High cholesterol	11.3	–	–
Irritable bowel syndrome	9.9	–	–
Unclassifiable	10.5	–	–
Hypothyroidism	9.5	–	–
Hay fever	8.6	–	–
Migraine	6.5	–	–
Gastric reflux	7	–	–
Medications (%)			
Fish oil, including cod liver oil	16	28.5	8.57 × 10^−14^
Blood pressure medication	40.1	0.2	0
Cholesterol lowering medication	19.4	0.8	0

Median values and the interquartile range (IQR) are reported for numerical variables and percentages for categorical variables. Mann-Whitney U test and chi-square test of independence with Yates continuity correction were performed on numerical and categorical data, respectively, to compare the differences in ME/CFS against healthy controls.

a Missing values percentages are not presented.

### Metabolite genome-wide association studies with covariate multi-phenotype studies method

In this study, we performed a quantitative GWAS on 135 NMR metabolic biomarkers, including lipoprotein subclasses, lipids, fatty acid ratios, and low molecular weight metabolites (Data S1) in both ME/CFS and HCs. We used the Covariate Multi-phenotype Studies (CMS) method,^26^ which has been optimized for high-dimensional phenotypic screening and increased effective power (See STAR Methods). There were 87 biomarkers that were significantly different between ME/CFS and HCs at the Benjamini-Hochberg adjusted *p* < 0.05 (Data S2); however, all 135 biomarkers were retained in the mGWAS screen to identify overlapping variants that may regulate metabolic biomarker levels.

Association tests were adjusted for confounding factors including age, sex, fasting time, body mass index (BMI), medication (cholesterol lowering and blood pressure), supplements (fish oil), and comorbid conditions (hypertension, asthma, depression, osteoarthritis, high cholesterol, irritable bowel syndrome, unclassifiable, hypothyroidism, hay fever, migraine, and gastric reflux). SNPs were grouped into independent regions based on linkage disequilibrium (LD),^27^ where the SNP with the lowest *p*-value was appointed to represent each region.^18^ There was a total of 112 SNP region-biomarker associations in the ME/CFS cohort at *p* < 1.61 × 10^−9^ (Data S3), using the standard genome-wide significant threshold of *p* < 5 × 10^−8^, which was adjusted for the number of effective biomarker tests (Figure S1A).

The CMS method identified 4.3 times more SNP region-biomarker associations than standard linear regression (STD) (Figure S1D). No hidden population structure was identified through quantile-quantile (QQ) plots and the genomic inflation factor (λ), where λ_CMS_ = 0.83 and λ_STD_ = 1.0 (Figure S1B). Regression coefficients of significant SNPs identified by CMS and STD showed overall good correlation (Pearson’s r = 0.990), indicating minimal bias from including additional proxy covariates (Figure S1C).

### Discovery of unique single nucleotide polymorphisms in the myalgic encephalomyelitis/chronic fatigue syndrome cohort

**Figure 1:**
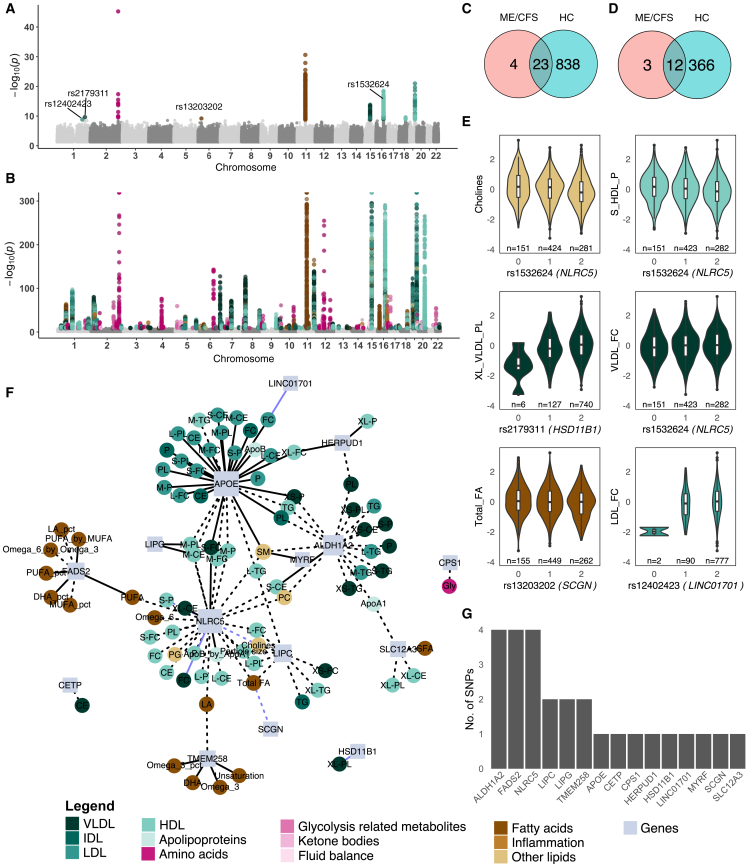
Overview of the SNP region-metabolite associations identified by the CMS method (A and B) Manhattan plots of mGWAS results for A) ME/CFS (*n* = 875) and B) HC (*n* = 36,033). (C and D) Significant SNPs (*p* < 1.61 × 10^−9^ and *p* < 1.31 × 10^−9^ for ME/CFS and HC, respectively) are colored according to their biomarker group (See legend). Venn diagrams show overlapping C) SNPs and D) genes between ME/CFS and HC. (E) Violin plots of inverse rank normalized metabolite levels by genotype of four significant SNPs found in ME/CFS only (rs1532624, rs2179311, rs13203202, rs12402423). Genotypes are coded as 0 = homozygous minor allele, 1 = heterozygote and 2 = homozygous major allele. Boxes show the interquartile range, the horizontal line indicates the median, and the points represent outliers. The number of individuals (n) in each genotype group is shown below the plots. (F) Network graph displays significant SNP region-biomarker associations in ME/CFS. Circular nodes represent biomarkers, square nodes represent genes; solid edges represent positive associations between SNP and biomarker, dashed edges represent negative associations, and cornflower blue edges represent associations unique to the ME/CFS cohort. (G) Bar chart shows the number of significant SNPs identified for each gene for ME/CFS. Genes shown here were prioritized based on functional impact, followed by proximity.

We identified 112 significant genetic associations in ME/CFS, across 8 biomarker groups including high-density lipoprotein (HDL) (*n* = 37), fatty acids (*n* = 19), low-density lipoprotein (LDL) (*n* = 19), very low-density lipoprotein (VLDL) (*n* = 17), other lipids (*n* = 9), IDL (*n* = 6), apolipoproteins (*n* = 4) and amino acids (*n* = 1) (Data S3). There was a total of 27 significant SNPs that mapped to 15 genes (Figures 1A–1G). Three of those SNPs had not been previously reported in the NHGRI-EBI GWAS catalog^28^ or were significantly associated in HCs. For rs2179311 (minor allele frequency, MAF 7.94%), an intronic variant proximal to *CAMK1G* was associated with phospholipids in extra-large VLDL (XL-VLDL-PL) (β = −0.19, *p* = 2.26 × 10^−10^). Functional analysis using Open Targets Genetics’ Variant-to-Gene (V2G) pipeline^29^ suggested *HSD11B1* as a candidate gene. For rs13203202 (MAF 44.0%), an intronic variant upstream of *CARMIL1* was associated with total fatty acids (β = −0.08, *p* = 6.63 × 10^−10^), and was predicted to affect *SCGN*. For rs12402423 (MAF 5.4%), an intronic variant of a long intergenic non-coding RNA, *LINC01701* was associated with free cholesterol in LDL (LDL-FC) (β = 0.13, *p* = 1.47 × 10^−9^). One intronic variant, rs1532624, downstream of *CETP* (and predicted to affect *NLRC5*) was significantly associated with total cholines (β = −0.17, *p* = 2.59 × 10^−14^), concentration of small HDL particles (S-HDL-P) (β = −0.14, *p* = 8.49 × 10^−14^), and VLDL-FC (β = 0.062, *p* = 2.48 × 10^−10^). While that SNP-biomarker association was not significant in HC, both *CETP* and *NLRC5* were perturbed in HCs via seven other SNPs (Data S4). Furthermore, the associations with the greatest functional impact determined by Ensembl Variant Effect Predictor (VEP)^30^ in ME/CFS included rs1047891 (MAF 31.2%), a missense variant of *CPS1* with glycine (β = −0.62, *p* = 1.93 × 10^−40^), and rs174535 (MAF 35.1%), a synonymous variant of *MYRF* with sphingomyelin (β = 0.06, *p* = 3.07 × 10^−12^). Both associations were also present in HCs (Data S4).

### Shared genetic associations in myalgic encephalomyelitis/chronic fatigue syndrome and healthy controls

The HCs yielded 4,114 significant SNP-region metabolite associations, including all 135 biomarkers across 861 SNPs, mapping to 366 genes at *p* < 1.51 × 10^−9^ (Figure 1B and Data S4; Figure S2). There were two stop-loss variants: rs328 (MAF 10.3%) of *LPL* associated with 23 different biomarkers; and rs5888 (MAF 48.9%) of *SCARB1* associated with 6 biomarkers. In total, 23 SNPs (mapping to 12 genes) overlapped between ME/CFS and HC, while 838 SNPs (366 genes) were significant only in HC (Figures 1C and 1D). The overlapping significant genes were involved in the urea cycle (*CPS1*), lipid metabolism (*APOE*, *CETP*, *LIPC*, *LIPG*), fatty acid metabolism (*FADS2*), glycosylation (*TMEM258*), innate immunity (*NLRC5*, *HERPUD1*), solute transportation (*SLC12A3*), retinoic acid synthesis (*ALDH1A2*), and signal transduction (*MYRF*). These shared associations likely reflect common genetic determinants of metabolic phenotypes in the general population, some of which may intersect with, but are not exclusive to, the pathophysiological processes in ME/CFS.

### Myalgic encephalomyelitis/chronic fatigue syndrome metabolite genome-wide association studies single nucleotide polymorphisms in previous case-control genome-wide association studies

We cross-referenced the 27 significant ME/CFS mGWAS SNPs against seven case-control GWASs (Data S5). None of the four unique SNPs reached a genome-wide significance in any of the case-control studies (Table 2). However, rs13203202 (*SCGN*) was significant for five studies, while rs2179311 (*HSD11B1*) was significant in one study at *p* < 0.1. For the generic SNPs; rs1047891 (*CPS1)* was significant in the DecodeME study^17^ and rs102274, rs102275, rs1535, rs174535, rs174550 (*TMEM258*, *FADS2*, *MYRF*, and *ALDH1A2,* respectively) were significant in the Million Veteran Program study^31^ at *p* < 0.05 (Data S5).

**Table 2:** Comparison of unique ME/CFS mGWAS SNPs with previous case-control GWASs

SNP	Prioritised gene	DecodeME^17^ (*n* = 15,579)		Million Veteran Program^31^ (*n* = 3,891)		Dönertaş et al.^32^ (*n* = 2,092)		GeneATLAS^33^ (*n* = 2,017)		Pan-UKB^34^ (*n* = 1,882)		Neale lab∗ (*n* = 1,659)		Zhou et al.^35^ (*n* = 593)	
		Effect (β)	p	Effect (β)	p	Effect (β)	p	Effect (β)	p	Effect (β)	p	Effect (β)	p	Effect (β)	p
rs12402423	*LINC01701*	2.50 × 10^−5^	0.999	0	1.000	1.14 × 10^−4^	0.71	9.07 × 10^−5^	0.776	5.77 × 10^−3^	0.9380	1.76 × 10^−5^	0.961	−0.033	0.800
rs2179311	*HSD11B1*	6.40 × 10^−4^	0.978	9.95 × 10^−3^	0.848	2.85 × 10^−4^	0.23	4.00 × 10^−4^	0.120	−0.102	**0.0923**	−3.24 × 10^−4^	0.269	0.030	0.783
rs13203202	*SCGN*	−8.94 × 10^−3^	0.478	0.041	**0.087**	−2.54 × 10^−4^	0.06	−2.47 × 10^−4^	**0.080**	0.064	**0.0547**	2.97 × 10^−4^	**0.065**	−0.016	0.790
rs1532624	*NLRC5*	0.014	0.259	0.025	0.278	−1.83 × 10^−4^	0.18	−1.87 × 10^−4^	0.190	0.036	0.2755	1.62 × 10^−4^	0.314	3.87 × 10^−3^	0.947

The studies selected all performed a case-control ME/CFS GWAS using genotype data in a European population. P-values <0.1 are bolded. Table for all 27 SNPs found significantly associated with a biomarker in ME/CFS can be found Data S5. Additional study information can be found in Table S1. The Neale lab summary statistics were downloaded from http://www.nealelab.is/uk-biobank/.

There are several reasons for the lack of replication of our mGWAS SNPs in the case-control studies. First, phenotypic heterogeneity across cohorts is substantial. DecodeME applied stringent clinical criteria (either Canadian Consensus Criteria^36^ or National Academy of Medicine guidelines^37^), and the Million Veteran’s Program defined cases via PheCode 798.1 derived from ICD-10 codes. Reliable ME/CFS case ascertainment in the UKB requires concordant evidence across interview, questionnaire, and ICD-10,^38^ which may reduce sample size and power. However, most of the UKB studies, including ours, used only the interview label. Additionally, comorbidity burden and disease severity vary within and between cohorts. Secondly, study-level methods differ, including genetic quality controls and analytical methods (Table S1). Despite five of seven studies using the same UKB resource and genotype array, there were inconsistencies in effect size directions across multiple SNPs. Furthermore, case-control GWAS assume a single marginal effect per variant; therefore, any subgroup specific effects may be attenuated. If genetic effects act only in small endotypes or biomarker effects are modest and noisy, both SNP-disease and SNP-biomarker associations can go undetected.

### Genetic effect sizes vary between myalgic encephalomyelitis/chronic fatigue syndrome and healthy controls

We hypothesize that any altered regulatory mechanisms from potential disease-specific or environmental factors would be reflected through different genetic effect sizes. To compare effect sizes between ME/CFS and HCs, which both have unequal sample sizes, we first applied a winner’s curse adjustment using a bootstrap resampling method to correct for any inflated effect sizes in the smaller ME/CFS cohort (Figure S3).^39^

**Figure 2:**
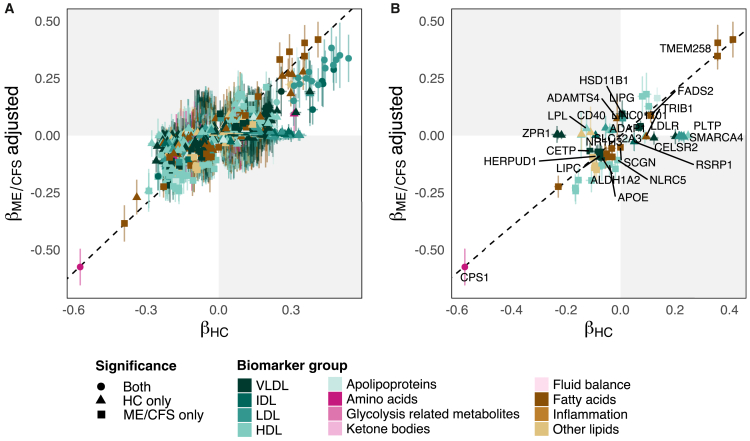
Genetic effect sizes between ME/CFS and HCs show low correlation (A and B) Scatterplots of the effect sizes (β) for (A) all significant associations found in ME/CFS and HC (Pearson’s r = 0.63, *p* < 2.20 × 10^−16^), and (B) the 65 associations that had significantly different effect sizes based on heterogeneity testing. The adjusted effect sizes for the ME/CFS cohort are shown, which have been corrected with bootstrap resampling. Cross-hairs represent the 95% confidence interval for each effect size. Colored symbols represent biomarker groups, and shapes represent significance as stated in the legend.

We observed moderate correlation between the adjusted ME/CFS and HC effect sizes of the 4,053 significant SNP-biomarker associations across both cohorts (Pearson’s r = 0.63, *p* < 2.20 × 10^−16^) (Figure 2A). When stratified by biomarker group, ketone body associations between ME/CFS and HC were the least correlated (r = 0.51, *p* = 0.09), followed by VLDL (r = 0.58, *p* = 2.35 × 10^−116^), and other lipids (r = 0.61, *p* = 1.12 × 10^−24^) (Figure S4). At a global level, if the biological processes in ME/CFS and HCs were operating at the same efficiency, the effect sizes would be expected to have a higher correlation.

To identify specific SNP-biomarker associations with different effect sizes, we performed heterogeneity tests using METAL,^40^ a meta-analysis tool for GWAS summary statistics. We found 65 genetic associations with significantly different effect sizes at Bonferroni adjusted *p* < 1.23 × 10^−5^ (Figure 2B and Data S6). The I^2^ statistic, which quantifies the proportion of total variation attributable to between-group heterogeneity, ranged from 94.8% to 98.6%, indicating substantial differences in genetic effects. Most of the SNP-biomarker pairs were significant only in either ME/CFS (*n* = 38) or HC (*n* = 26) cohorts, except for one, rs1047891 (*CPS1*)—glycine, which was a top association in both cohorts. We identified a subset of associations potentially involved in the immunogenetic regulation of lipid metabolism including rs10951261—ApoB (β_ME/CFS_ adjusted = −0.02, β_HC_ = 0.02, I^2^ *p* = 1.60 × 10^−6^), rs4752983—concentration of large HDL particles (L-HDL-P) (β_ME/CFS_ adjusted = 0.02, β_HC_ = −0.04, I^2^ *p* = 3.35 × 10^−6^), and rs10432735—cholesteryl esters in large LDL (L-LDL-CE) (β_ME/CFS_ = 0.03, β_HC_ = −0.05, I^2^ *p* = 3.59 × 10^−6^), with the three variants mapping to *ADAP1*, *NR1H3,* and *CD40,* respectively. These results suggest that genetic effects on metabolite levels may differ between patients with ME/CFS and healthy individuals, supporting the use of biomarker phenotypes to uncover regulatory variation that may be overlooked in binary case-control designs.

We also compared the MAF and the genotype distributions of all significant SNPs found in both ME/CFS and HCs to identify potential overrepresented variants in ME/CFS (Data S7). A z-proportional test showed no significant differences in MAFs between the two populations at the Bonferroni adjusted threshold of *p* < 5.88 × 10^−5^. However, only two SNPs: rs4968318, a missense variant of *EFCAB13* (MAF_ME/CFS_ = 42.7%, MAF_HC_ = 39.1%, *p* = 0.03) and rs1260333, an intronic variant distal to *GCKR* (MAF_ME/CFS_ = 46.2%, MAF_HC_ = 42.8%, *p* = 0.04) were significant at the unadjusted threshold of *p* < 0.05. We then performed a chi-square test to determine whether genotype frequencies (homozygous major, heterozygous, homozygous minor) were different between ME/CFS and HC. Again, no SNPs were significant at the Bonferroni adjusted *p*-value; however, 42 SNPs were significant at the unadjusted significance threshold (Data S7). If significant differences in MAFs or genotype distributions had been observed in the ME/CFS cohort, it could have indicated a potential link between specific alleles and ME/CFS itself. While our findings suggest a few potential SNPs that may contribute to dysregulated pathways in ME/CFS, the effects appear to be small and require larger sample sizes to detect with greater certainty.

### Decomposition of summary statistics and pathway enrichment

Metabolites function as substrates, intermediates, or end-products in various biochemical pathways, making it challenging to understand all the different roles they play and the downstream effects of their interactions with other biological molecules. To uncover the full spectrum of biological pathways in which these metabolomic biomarkers are involved, we applied the Decomposition of Genetic Associations (DeGAs) framework to ME/CFS mGWAS summary statistics.^24^ DeGAs reduces high-dimensional association data into orthogonal components that capture the shared genetic architecture driving phenotypic variation (in our case, biomarker levels). This unsupervised, exploratory approach enables the identification of coordinated genetic effects, generating hypotheses about latent biological processes that may contribute to ME/CFS pathophysiology. Additionally, DeGAs incorporates both significant and sub-significant associations that did not pass genome-wide significance to reveal subtle, distributed genetic patterns that a conventional GWAS threshold may overlook.^41^

We first employed a variant filter (*p* < 0.001, standard error < 0.08) to ensure reliable effect estimates, followed by truncated singular value decomposition (TSVD) on a sparse Z-scored summary statistic matrix with 135 biomarkers and 48,043 variants. The genetic associations, including non-coding and coding variants, were decomposed into 24 components, cumulatively explaining 55.1% of the variance in the original DeGAs matrix (Figures S5 and S6; Table S2).

**Figure 3:**
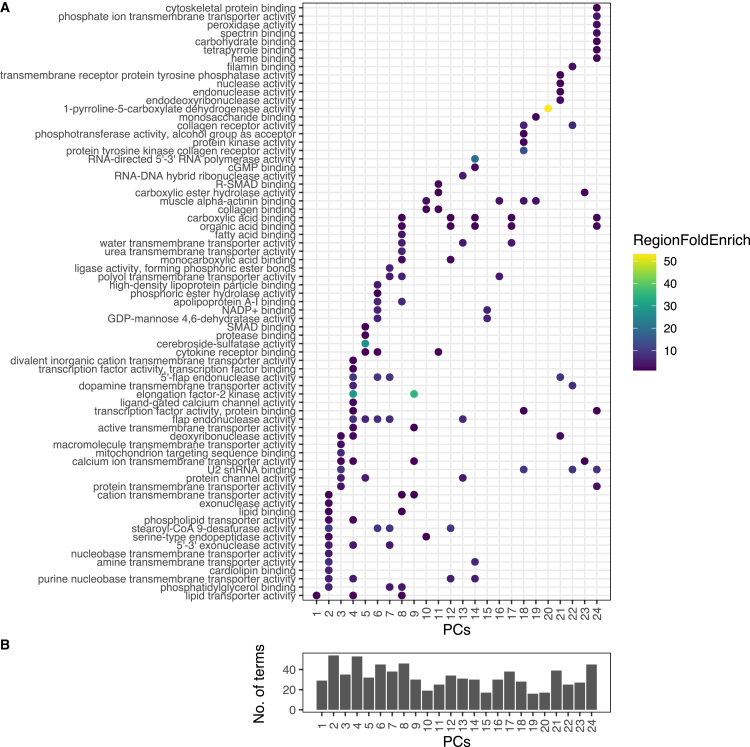
Enriched GO molecular functions in 24 latent components derived from summary statistics (A) Ninety out of the 150 GO molecular functions that were enriched (Bonferroni adjusted P_enrichment_<0.05) in five or fewer components are shown in this figure. The color of the points represents the fold enrichment for the genomic region. (B) Total number of molecular functions enriched for each component.

Functional annotation of the top 5000 variant contributions (explaining 62–88% of the genetic variation) for each component was performed using GREAT.^42^^,^^43^ We identified a total of 75 enriched Ensembl genes, 287 GO biological processes, 54 GO cellular components, 115 GO molecular functions (Figure 3) and 52 human phenotypes (Data S8). There were five molecular functions that appeared across all 24 components, including apolipoprotein binding, lipoprotein lipase activity, monoamine transmembrane transporter activity (which identified genes that were not significant in summary statistics such as *SLC18A1*), triglyceride binding, and triglyceride lipase activity. The repeated enrichment of these molecular functions across components suggests that disturbances in lipid metabolism and monoaminergic signaling may represent upstream processes contributing to the diverse downstream dysregulation observed in ME/CFS.

There were several components that were characterized by clusters of similar molecular functions and biological processes (Data S8). Component 5, which mostly captured VLDL variation (51.0%) (Table S2) was enriched in both pro-inflammatory and anti-inflammatory pathways, involving leukocyte-mediated immunity (P_enrichment_ = 4.15 × 10^−7^) and neutrophil degranulation (P_enrichment_ = 8.3 × 10^−7^) through *NLRC5*, *LPL*, and *AQP9* (Data S8). Additionally, glycosaminoglycan binding (P_enrichment_ = 2.88 × 10^−8^) and SMAD binding (P_enrichment_ = 2.90 × 10^−4^) also point to component 5’s involvement in specific signaling pathways, such as those mediated by TGF-β, which are crucial for balancing immune activation and suppression.

Component 6, capturing 82.5% of the biomarker variation through fatty acids, was enriched in linoleoyl-CoA desaturase activity (P_enrichment_ = 1.11 × 10^−6^) and omega-6 fatty acid desaturase activity (P_enrichment_ = 5.59 × 10^−8^). Furthermore, component 18, which captured 41.8% of the biomarker variation with VLDL, was significantly enriched in protein tyrosine kinase collagen receptor activity (P_enrichment_ = 1.11 × 10^−6^) and muscle alpha-actinin binding (P_enrichment_ = 1.49 × 10^−6^) through *DDR1*. DDR1, a collagen receptor, is critical for extracellular matrix interactions and cytoskeletal integrity. Dysregulation in these pathways may impair mechanotransduction, potentially contributing to the heightened sensitivity to mechanical stress and connective tissue problems.^44^

## Discussion

Circulating blood lipoprotein, lipid, and metabolite levels reflect an individual’s metabolic homeostasis, with deviations due to genetic or environmental factors potentially indicating disease. In this study, we aimed to identify gene pathways that contribute to the different ME/CFS metabolic profiles using the CMS method, which has been optimized to screen hundreds of continuous metabolic biomarkers.^18^ The mGWAS identified 2 novel SNPs (rs2179311, rs13203202) functionally mapping to *HSD11B1* and *SCGN,* respectively, where *HSD11B1* was previously implicated in ME/CFS via a different SNP (rs846906) from a candidate gene study.^45^ There were also 12 significant genes overlapping with HC and 366 genes that were found in HC only. These preliminary findings suggest the presence of both disease-specific and non-disease-related genetic factors that may influence to the dynamic fluctuations in biomarker levels observed in patients with ME/CFS.^46^

Our study suggests a possible genetic contribution to the neuroendocrine abnormalities observed in ME/CFS,^47^ implicating *HSD11B1* and *SCGN*, which have roles along the hypothalamic-pituitary-adrenal (HPA) axis. *HSD11B1* encodes 11β-hydroxysteroid dehydrogenase type 1, an enzyme that reversibly converts cortisone and cortisol in glucocorticoid target tissues such as adipose and liver.^48^ Since active cortisol is essential for metabolic regulation,^49^ endocrine function, immune modulation, and stress-related responses,^50^ genetic variation in *HSD11B1* could plausibly perturb these processes, which have all been highlighted in ME/CFS pathophysiology.^2^

Our interpretation remains cautious, considering mixed prior cortisol findings in ME/CFS. Altered cortisol circadian rhythms, blunted pituitary adrenocorticotropic hormone responses, and enhanced negative feedback regulation demonstrated by dexamethasone and prednisolone suppression tests have been reported, which are consistent with mild hypocortisolism.^51^ However, these results are largely based on circulating or excreted cortisol measures in plasma, saliva or urine, rather than direct assessments within *HSD11B1*-rich target tissues. Future work is required to clarify this distinction, as tissue-specific cortisol regeneration can differ from systemic levels and potentially obscure local pathophysiology.

We also identify *SCGN*, encoding secretagogin, as a gene of interest for ME/CFS. Secretagogin is a stress-responsive calcium-binding protein enriched in neuroendocrine tissues,^52^ including pancreatic β-cells, where it regulates insulin secretion,^53^ and in the hypothalamus, where it promotes corticotropin-releasing hormone release and neuronal stability under stress.^54^ Although *SCGN* has not been previously linked to ME/CFS, its role in coordinating neuroendocrine and metabolic responses to physiological stress^55^^,^^56^ positions it as a compelling candidate for underlying the disease’s dysregulated stress response. Secretagogin may also help explain clinical features such as autonomic instability and sensory hypersensitivity.

Among the genetic associations showing significant changes in effect sizes was *NR1H3*, which encodes liver X receptor alpha (LXRα). LXRα is highly expressed in the liver and gastrointestinal tract, where it regulates lipid homeostasis by controlling cholesterol efflux and bile acid synthesis,^57^ and suppresses pro-inflammatory macrophage activity.^58^ The dysregulation of these processes is consistent with previous reports of altered cholesterol and bile synthesis^11^ and chronic activation of the innate immune system in ME/CFS.^59^ The decomposition analysis of summary statistics also revealed enrichment in both pro- and anti-inflammatory pathways, in which the simultaneous activation of these opposing immune processes in monocytes has also been described.^10^ Additionally, ME/CFS individuals in the UK Biobank exhibited elevated glycoprotein acyls,^8^ a composite biomarker for systemic inflammation, known to be associated with various inflammatory conditions.^60^

The association of *CPS1* with glycine (significant in both ME/CFS and HC) highlights the role of the liver in ammonia detoxification via the urea cycle. *CPS1* encodes the enzyme catalyzing the first rate-limiting step of the urea cycle, which relies on N-acetylglutamate (NAG), an allosteric activator that is indirectly synthesized from glycine. Metabolomic studies in ME/CFs have reported altered levels of urea cycle-related metabolites, including reduced ornithine^9^ and citrulline,^5^ suggesting inefficiencies in ammonia detoxification. Although glycine has not been identified as a potential biomarker in ME/CFS,^46^ it plays a critical role in nitrogen balance through the glucose-alanine cycle and one-carbon metabolism, the latter being implicated in NADPH production^11^ and redox imbalance.^61^

Lipoproteins transport lipids through the bloodstream, supplying energy, nutrients, and structural components for lipid rafts, which are dynamic microdomains on cell membranes that facilitate neurotransmitter signaling and receptor activity.^62^ These rafts rely on a balanced lipid composition of cholesterol and sphingomyelin to maintain membrane stability and fluidity. The decreased sphingomyelin observed in ME/CFS can disrupt these lipid rafts,^8^ impairing the function of membrane-bound proteins such as the vesicular monoamine transporter 1 encoded by *SLC18A1* (found to be enriched in the decomposition and enrichment analysis), which is responsible for efficiently loading serotonin, dopamine, and norepinephrine into synaptic vesicles. Disruptions in lipid raft integrity could reduce neurotransmitter availability, contributing to the cognitive dysfunction, memory impairments, and mood disturbances observed in ME/CFS.

The *MYRF* gene was associated with sphingomyelin in both ME/CFS and HC cohorts, showing additional lipid associations exclusively in HC. Loss of the MYRF transcription factor disrupts myelin production, a process linked to demyelination. In ME/CFS, demyelination has been observed through magnetic resonance imaging studies,^63^ and plasma catalytic antibodies analyses.^64^

Furthermore, our study supports the idea that ME/CFS is driven by many genetic variants with small individual effects, and when aggregated, disrupt metabolic pathways.^15^ While these small-effect variants may be common in the general population, specific SNP clusters can exert a combinatorial effect, resulting in bottlenecks in key biological processes. SNPs influencing metabolite levels in HCs provide valuable insights into baseline metabolic regulation, even in the absence of disease. Although the greater number of associations in HC can be attributed to a larger sample size, many were linked to genes involved in metabolic pathways critical for ATP production, which were not observed in ME/CFS. These include genes associated with amino acid metabolism (*SLC1A4*, *BCAT2*), glycolysis (*PFKP*, *PFKFB2*, *GCKR*), and ketone metabolism (*OXCT1*, *ACSS2*). The identification of these pathways in HCs underscores their role in maintaining energy homeostasis and highlights potential disruptions that may occur in ME/CFS. We see in the effect size correlations (Figure S4) that many ME/CFS associations were attenuated (although with large 95% confidence intervals), particularly for glycolysis related metabolites, ketone bodies, and other lipids. Further investigation into how these pathways differ between healthy individuals and patients with ME/CFS could provide crucial insights into the metabolic bottlenecks contributing to the disease’s pathophysiology. Such bottlenecks may create an inability to compensate for or adapt to metabolic stress, resulting in the systemic dysfunction observed in ME/CFS.

While these findings offer compelling hypotheses regarding the underlying mechanisms in ME/CFS, direct experimental validation is required to progress them into a confirmatory role. These could include gene expression studies in specific tissues, cells, or organoids, such as the liver, brain, and adipose, to validate the roles of *HSD11B1*, *SCGN*, *NLRC5*, *NR1H3*, *SLC18A1*, *MRYF,* and so forth, in their respective pathways. Additionally, isotopically labeled experiments tracking energy metabolism and ammonia dynamics across different cell types could provide deeper insights into how these processes interact in ME/CFS.^65^

Overall, this study shows that biomarkers can help bridge genetic background with pathophysiological mechanisms, serving as both endpoints and mediators for health and disease. The mGWAS approach links genetic variants to perturbed metabolic traits, offering a third “lens” through which to explore the genomic and metabolic architecture of ME/CFS. This integrative perspective not only enhances our understanding of the biological processes involved but also strengthens the potential for discovering actionable biomarkers and therapeutic targets for ME/CFS subgroups.

### Limitations of the study

One key limitation is the absence of robust methods to identify causal SNPs for ME/CFS. Our results are associational: they highlight variants linked to disease-relevant metabolic pathways disrupted in ME/CFS and cannot confirm causality. To strengthen causal inference, future work will apply Mendelian randomization (MR), which uses genetic variants (often SNPs from well-powered and credible GWAS) as instrumental variables to assess the causal effect of an exposure (e.g., metabolite levels) on an outcome (e.g., ME/CFS).^19^^,^^66^ We will also consider multivariable and stratified MR to assess potential modifiers, such as sex, menopausal status, insulin resistance, infection history, disease duration, and symptom severity, and, where instrument strength permits, mediation MR analyses to investigate indirect pathways.^67^

Another limitation of this study is the potential presence of “winner’s curse,” particularly due to the difference in sample sizes between the ME/CFS cohort (*n* = 875) and the healthy controls (*n* = 36,033). Winner’s curse refers to the inflation of effect sizes for significant SNPs, especially when the sample size is small.^68^ This can lead to the overestimation of the true genetic effect in ME/CFS, making it difficult to replicate these findings in future studies. Given that the primary aim of this study was to screen for potential SNP-metabolite associations, we focused on identifying key associations and only applied an effect size correction when directly comparing ME/CFS effect sizes to HCs.

The UK Biobank is subject to a well-known volunteer bias, with participants generally representing a healthier subset of the population. This may limit the generalizability of findings and diminish the ability to detect biological disease signals. HCs were defined based on the absence of health problems at the time of assessment, without accounting for any conditions developed later, which could introduce heterogeneity. Similarly, the ME/CFS group was based on self-reported diagnosis without the clarification of the case criteria met, potentially resulting in a more diverse cohort than typically used in recent ME/CFS studies.

## Resource availability

### Lead contact

Further information and requests for resources should be directed to and will be fulfilled by the lead contact, Christopher Armstrong (christopher.armstrong@unimelb.edu.au).

### Materials availability

This study did not generate new unique reagents.

### Data and code availability

• This study uses data from the UK Biobank. Raw data can be accessed following approval of a research access application to the UK Biobank. The application number for this project is 79568. Complete summary statistics were uploaded to Mendeley Data: https://doi.org/10.17632/ch3mb4x8jk.1.
• This study did not generate any original code.
• Any additional information required to reanalyze the data reported in this article are available from the lead contact upon request.

## Acknowledgments

This research was approved by the UK Biobank under the application number 79568 and received funding from Open Medicine Foundation Australia and The Judith Jane Mason & Harold Stannett Williams Memorial Foundation, as managed by Equity Trustees. Analyses were performed on the UKB Research Analysis Platform and the High-Performance Computing system at The University of Melbourne. We thank the participants of the UK Biobank for their contribution to the resource, and researchers contributing to the returned datasets. D.B.A. was supported by The 10.13039/501100000925National Health and Medical Research Council of Australia (GNT1174405), and The Victorian Government’s Operational Infrastructure Support Program. E.K.S. was supported by the 10.13039/501100000925NHMRC (2024/GNT2036793), the VESKI Fair fellowship, and the research impetus grant from the University of Melbourne.

## Author contributions

Conceptualization: K.H. and C.W.A. Formal analysis, methodology, and software: K.H. and M.N. Resources: C.W.A., P.R.G., and D.B.A. Writing – original draft: K.H. Writing – review and editing: K.H., M.N., N.T., E.K.S., D.B.A., P.R.G., and C.W.A. Visualization: K.H. Supervision: C.W.A., P.R.G., and D.B.A. Funding acquisition: P.R.G. and C.W.A.

## Declaration of interests

Authors report no financial or non-financial competing interests.

## STAR★Methods

### Key resources table

**Table:** 

REAGENT or RESOURCE	SOURCE	IDENTIFIER
**Deposited data**		

Observational data	UK Biobank	https://www.ukbiobank.ac.uk/; Project #79568
ME/CFS mGWAS summary statistics	This paper	Mendeley data https://doi.org/10.17632/ch3mb4x8jk.1
Healthy control mGWAS summary statistics	This paper	Mendeley data https://doi.org/10.17632/ch3mb4x8jk.1
DecodeME^17^ ME/CFS case-control GWAS summary statistics	Original author’s own repository	https://osf.io/rgqs3/files
Verma et al.^31^ ME/CFS case-control GWAS summary statistics	GWAS catalog	GCST90479178
Pan-UKB ME/CFS case-control GWAS summary statistics	Pan-UKB	https://pan.ukbb.broadinstitute.org/
Dönertaş et al.^32^ ME/CFS case-control GWAS summary statistics	GWAS catalog	GCST90038694
Canela-Xandri et al.^33^ ME/CFS case-control GWAS summary statistics	GeneATLAS	http://geneatlas.roslin.ed.ac.uk/
Zhou et al.^35^ ME/CFS case-control GWAS summary statistics	GWAS catalog	GCST90436811
Neale lab ME/CFS case-control GWAS summary statistics	Neale lab	http://www.nealelab.is/uk-biobank/

**Software and algorithms**		

ukbnmr v2.2 (R package)	Ritchie et al.^69^	https://cran.r-project.org/web/packages/ukbnmr/
GenABEL v1.8 (R package)	Aulchenko et al.^70^	https://CRAN.R-project.org/package=GenABEL
plink v2.0	UK Biobank Research Analysis Platform Swiss Army Knife	PLINK (RRID:SCR_001757)
Picard LiftoverVcf v4.0.2.0	Broad Institute	https://gatk.broadinstitute.org/hc/en-us/articles/360037060932-LiftoverVcf-Picard
CMS	Gallois et al.^18^	https://gitlab.pasteur.fr/statistical-genetics/runCMS
winnerscurse v0.1.1 (R package)	Forde et al.^39^	https://github.com/amandaforde/winnerscurse
Open Targets Genetics	Ghoussaini et al.^29^	Open Targets Genetics Portal (RRID:SCR_021701)
VEP	McLaren et al.^30^	Variant Effect Predictor (RRID:SCR_007931)
METAL	Willer et al.^40^	https://genome.sph.umich.edu/wiki/METAL
Irlba v2.3.5.1 (R package)	Baglama et al.^71^	https://cran.r-project.org/web/packages/irlba/index.html
GREAT v4.0.4	McLean et al.^42^; Tanigawa et al.^43^	GREAT: Genomic Regions Enrichment of Annotations Tool (RRID:SCR_005807)
stats v4.1.3 (R package)	N/A	https://stat.ethz.ch/R-manual/R-devel/library/stats/html/00Index.html

**Other**		

liftOver WDL script	UK Biobank Research Analysis Platform (DNAnexus)	https://github.com/dnanexus-rnd/liftover_plink_beds
UCSC Browser	Raney at al.^72^	https://genome.ucsc.edu/index.html
DeGAs	Tanigawa et al.^24^	https://github.com/rivas-lab/public-resources/tree/master/uk_biobank/DeGAs
NHGRI-EBI GWAS Catalog	Sollis et al.^28^	https://www.ebi.ac.uk/gwas/

### Experimental model and study participant details

#### Study population

In this study, UKB ME/CFS participants were identified through self-reported clinical diagnosis via the verbal interview^73^^,^^74^ (data field: 20002, illness code: 1482). Diagnoses and baseline characteristics were extracted from the initial assessment visit, aligning with the first biological sample donation used for (phase 1 and 2) NMR metabolomics profiling. There were 2,161 participants who reported a clinical ME/CFS diagnosis at baseline, in which 1,194 participants had available NMR metabolomics data. Participants that did not report any cancer or non-cancer medical conditions at baseline were selected as HCs. The final study sample included 875 ME/CFS participants (median age of 56 years, 76% females) and 36,033 HCs (median age of 54 years, 53% females) (Table 1) who also passed genetic sample quality control (QC) which involved filtering for White British ancestry (See method details). The ME/CFS cohort had a greater proportion of females compared to controls, reflecting known epidemiological patterns. Although sex was included as a covariate in the mGWAS, we did not perform extensive sex-stratified analyses and highlight this as a future direction. Participants who have requested to be withdrawn were removed from the study. All participants provided written informed consent, and ethics approval was obtained from the Northwest Multi-centre Research Ethics Committee. The current project was approved under UKB Project #79568.

### Method details

#### NMR biomarker data

The metabolic biomarkers were quantitated using high-throughput NMR spectroscopy by the Nightingale Health platform.^75^ This dataset includes various bulk measurements of lipid classes. To reduce the redundancy of downstream association testing and avoid inflating the multiple testing burden due to overlapping measurements, 135 biomarkers were selected (including 106 non-derived biomarkers, 18 composite biomarkers, and 11 ratios) from the total 251 biomarkers available on the UKB. Prior to CMS analysis, biomarker data were processed by removing technical variation correcting for sample preparation time, shipping plate well, and spectrometer batch effects,^69^ followed by an inverse normal rank transformation to reduce the effects of outliers.^18^^,^^70^

#### Sample and variant quality control

Participants were filtered for White British ethnicity and related individuals were removed (i.e., those not included in the UKB computation of genetic principal components). Genotype data (sequenced on two different UK Biobank arrays: Applied Biosystems UK BiLEVE Axiom Array and UK Biobank Axiom Array^73^) were remapped to the GRCh38 reference genome prior to quality control, which involved filtering variants with minor allele frequency (MAF) < 5%, missing rate <10% or P-value for Hardy Weinberg equilibrium <1 × 10^−9^. The final number of SNPs were 360,422 and 364,379 for the ME/CFS and HC cohorts respectively.

#### Metabolite GWAS

mGWAS was performed using the Covariate Multi-phenotype Studies (CMS) algorithm (Equation 1) in an established pipeline by Gallois et al.^18^ First, pairwise marginal effects were calculated between the outcome variable (*Y*_*m*_, representing biomarker *m*), genetic variant (*G*), and covariates (*Y*_*l*_, where *l* is a subset of potential covariates excluding *m*) using standard linear regression (STD). Covariates that were associated with the genetic variant were filtered out through multivariate analysis of variance (MANOVA) and conditional association tests.^26^ The final linear regression was conducted with the selected covariates and adjusted for confounding variables (*C*), including age, sex, fasting time, body mass index, medication (cholesterol lowering and blood pressure), supplements (fish oil) and comorbid conditions (hypertension, asthma, depression, osteoarthritis, high cholesterol, irritable bowel syndrome, unclassifiable, hypothyroidism, hay fever, migraine and gastric reflux). Confounders were selected from variables that may affect downstream biomarker levels and included if they were significantly different between ME/CFS, and HCs determined by Mann-Whitney U test or Chi-square test of independence (Table 1). The rationale behind CMS is to incorporate proxy variables that can explain the variation observed in the outcome variable, without it being associated to the genetic variant, as covariates. This reduces residual variance, thereby increasing the effective sample size.

(Equation 1) $$\begin{array}{c} Y_{m}∼\beta_{G}G+\beta_{C}C+\beta_{l}Y_{l} \end{array}$$

To reduce the risk of false positives from the additional covariates (*Y*_*l*_), we excluded biomarkers that were highly correlated (Pearson’s r > 0.9) prior to running analysis, which naturally removed all biomarkers in the same lipoprotein subclass as *Y*_*m*_. We also applied a pre-selection procedure according to the original manuscript that ranked the top 30 candidate covariates by Akaike Information Criteria and excluded any biomarkers that explained >70% of the variance observed in *Y*_*m*_.

#### Gene assignment of LD blocks

Post-GWAS processing was performed at the final step of the CMS pipeline.^18^ Briefly, results were summarised into 1,703 independent regions, based on a recombination map approximating linkage disequilibrium (LD) blocks.^27^ The most significant SNP within each region identified by either CMS or STD was designated to represent that block. Genes were assigned using three methods. The nearest gene to each SNP (within a maximum distance of 200 kb) was assigned using the hg38 genome assembly in the UCSC database.^72^ Variant annotation was performed with the Variant Effect Predictor (VEP),^30^ which prioritises gene assignments based on predicted functional consequences of variants, including their effects on protein structure, conservation scores and regulatory annotations. The Open Targets Genetics Variant-to-Gene (V2G) pipeline^29^ was also applied, which predicts gene associations by integrating functional genomics data including chromatin accessibility, expression quantitative trait loci, and promoter-enhancer interactions that assigns scores to tank genes based on their likelihood of being functionally affected by the SNP.

#### Decomposition of genetic associations

We followed the Decomposition of Genetic Associations (DeGAs) framework developed by Tanigawa et al.,^24^ and performed truncated singular value decomposition (TSVD) on a Z-scored summary statistics. Marginal effects with *p* > 0.001 or standard error > 0.0.08, according to author’s recommendations, were replaced with missing values (NAs).^41^ Subsequently, variants with NAs across all 135 biomarkers were filtered out. Remaining SNPs were summarised according to LD blocks and transformed into Z-scores so that biomarker vectors have a mean estimate of zero and unit variance. The final matrix was transposed to have 135 biomarker rows and 48,043 variant columns. We implemented Implicitly restarted Lanczos bidiagonalization algorithm^71^ to factorise the DeGAs matrix (W) with a pre-determined number of latent components (*k=24*) into a product of three matrices (Equation 2): an orthonormal matrix (*U*) composed of biomarker singular vectors, a diagonal matrix where the diagonal elements represent the singular values (*S*), and an orthonormal matrix (*V*) composed of the variant singular vectors, using the package irlba in R. Here, we set *k=24* as it explained over 50% of the variation in the DeGAs matrix, while remaining informative in the latter components.

(Equation 2) $$\begin{array}{c} W=USV^{T} \end{array}$$

Factor scores (Equation 3), contribution scores (Equation 4), and squared cosine scores (Equation 5) that were used to interpret latent components were calculated according to Tanigawa et al.^24^ Equations below show the calculations for biomarker *i*. To calculate scores for variant *j*, substitute matrix $U={\left(u_{i,k}\right)}_{i,k}$ with matrix $V={\left(v_{j,k}\right)}_{j,k}$.

(Equation 3) $$\begin{array}{c} F_{b}=US={\left(f_{i,k}^{b}\right)}_{i,k} \end{array}$$

(Equation 4) $$\begin{array}{c} {cntr}_{k}^{bio}\left(i\right)={\left(u_{i,k}\right)}^{2} \end{array}$$

(Equation 5) $$\begin{array}{c} {\cos_{i}^{2}}^{bio}\left(k\right)=\frac{{\left(f_{i,k}^{b}\right)}^{2}}{\sum_{k^{\prime }}{\left(f_{i,k^{\prime }}^{b}\right)}^{2}} \end{array}$$

#### Annotation of latent components with GREAT

The top 5,000 ranked variants based on contribution scores for each DeGAs component were analyzed with the Genomic Region Enrichment Analysis Toolset (GREAT v4.0.4).^42^^,^^43^ GREAT associates each non-coding variant with nearby genes by defining regulatory domains, which include both proximal (5.0 kb upstream, 1.0 kb downstream) and distal regions (up to 1000.0 kb) around the genes, thereby linking these variants to potential gene regulatory effects. The tool applies a binomial test to assess whether the observed association between the genomic regions and specific biological terms is greater than would be expected by chance. We tested each component variant set against the whole genome to obtain enriched gene ontology (GO) biological processes, GO cellular components, GO molecular functions, human phenotypes. Gene ontology terms were downloaded on 2024-07-29 and significantly enriched GO terms were identified using the Bonferroni corrected binomial *p*-value <0.05.

### Quantification and statistical analysis

#### GWAS multiple testing comparison adjustment

Principal components analysis was performed on the 135 biomarkers tested to determine the number of effective tests which were used to further adjust the standard genome-wide significant threshold of 5 × 10^−8^. We found 31 and 33 PCs accounted for 99% of the biomarker variation in ME/CFS and HC, setting the final significance threshold as *p* < 1.61 × 10^−9^ and *p* < 1.31 × 10^−9^ respectively. All *p*-values provided in this manuscript are presented as the raw unadjusted value.

#### Effect size adjustment

ME/CFS effect sizes were adjusted using bootstrap resampling implemented in the R package *winnerscurse.*^39^ Winner’s curse is a known bias in underpowered studies that leads to overestimation of effect sizes among significant findings. The correction was applied independently to the summary statistics for each biomarker. Both raw and adjusted effect sizes are reported, with any adjusted value denoted as such.

#### Statistical analyses

We performed a range of statistical analyses to assess differences in ME/CFS and HCs across genetic variables. Mann-Whitney U tests were performed to identify differences in metabolic biomarker concentrations between ME/CFS and HCs. Cochran’s Q-test for heterogeneity was performed to assess the differences in effect sizes and was implemented through METAL.^40^ A z-proportional test was performed to evaluate differences in MAFs. Chi-square test of independence was employed to assess whether the distribution of genotypes (homozygous major, heterozygous, and homozygous minor) differed between the groups. Statistical analyses, except for the Cochran’s Q-test were all performed in R using *stats* package.
