# Supplementary material for: Exploring a genetic basis for the metabolic perturbations in ME/CFS using UK biobank
Source: iScience. 2025 Dec 3;29(1):114316. doi: 10.1016/j.isci.2025.114316 (PMC12796752; doi:10.1016/j.isci.2025.114316)
Supplement: Document S1. Figures S1–S6 and Tables S1 and S2 [file mmc1.pdf]

iScience, Volume *29*

Supplemental information

Exploring a genetic basis for the metabolic perturbations in ME/CFS using UK biobank

Katherine Huang, Muhammad Muneeb, Natalie Thomas, Elena K. Schneider-Futschik, Paul R. Gooley, David B. Ascher, and Christopher W. Armstrong

Supplementary Figures

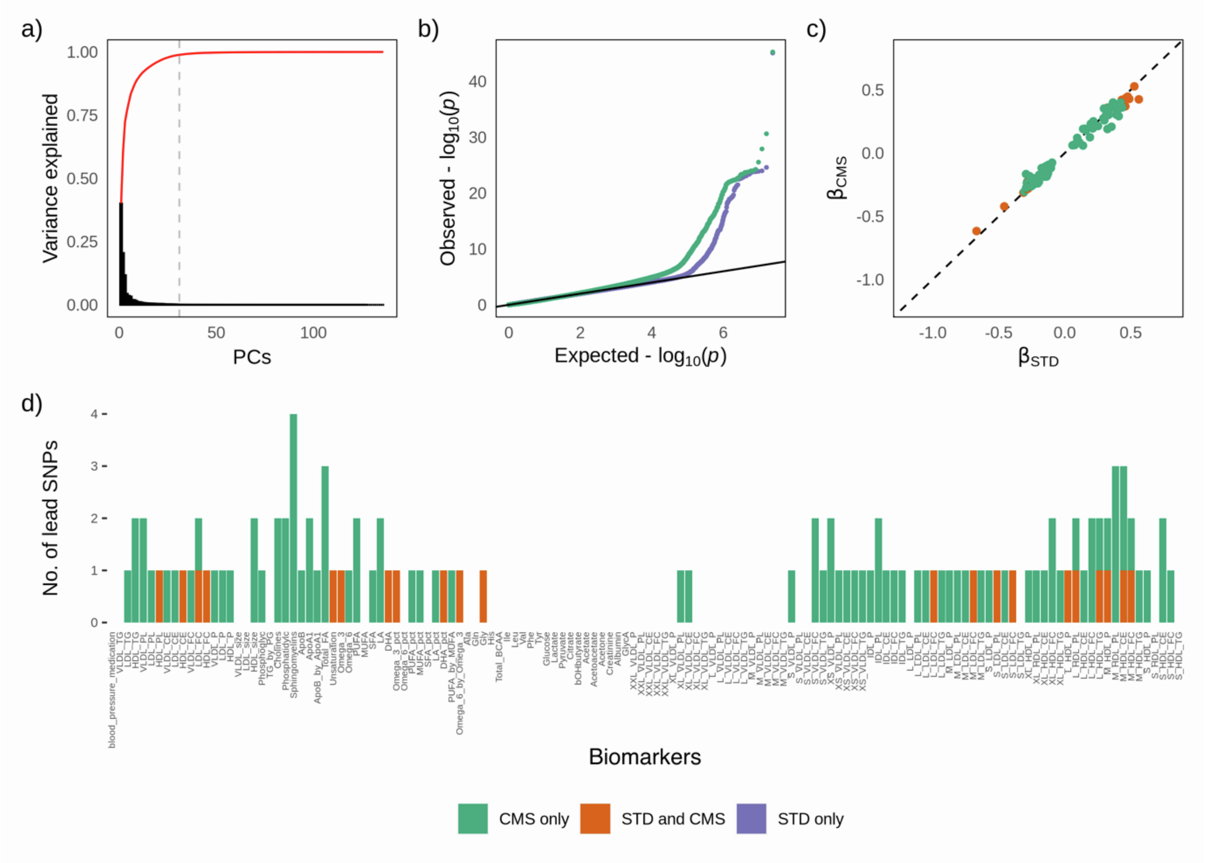

Supplementary Fig 1. GWAS quality control plots for ME/CFS cohort.
Principal components analysis was performed on the biomarker data. A) Scree plot shows the variance explained for each principal component (PC). The red curve represents the cumulative variance, and the grey dashed vertical line indicates the 31st PC (explaining 99% of the biomarker variance). B) QQ plots show the observed p-values for CMS (green) and STD (purple) where λ_{CMS}=0.83 and λ_{STD}= 1.0. C) Regression coefficients for significant SNP-regions estimated by CMS and STD are shown where Pearson's r=0.990. D) Comparison of the number of SNP region-metabolite associations found by CMS and STD. Significant SNPs at $p<1.61\times10^{-9}$ determined by CMS only are shown in green, both CMS and STD in orange, and none were found by STD only. Related to Figure 1.

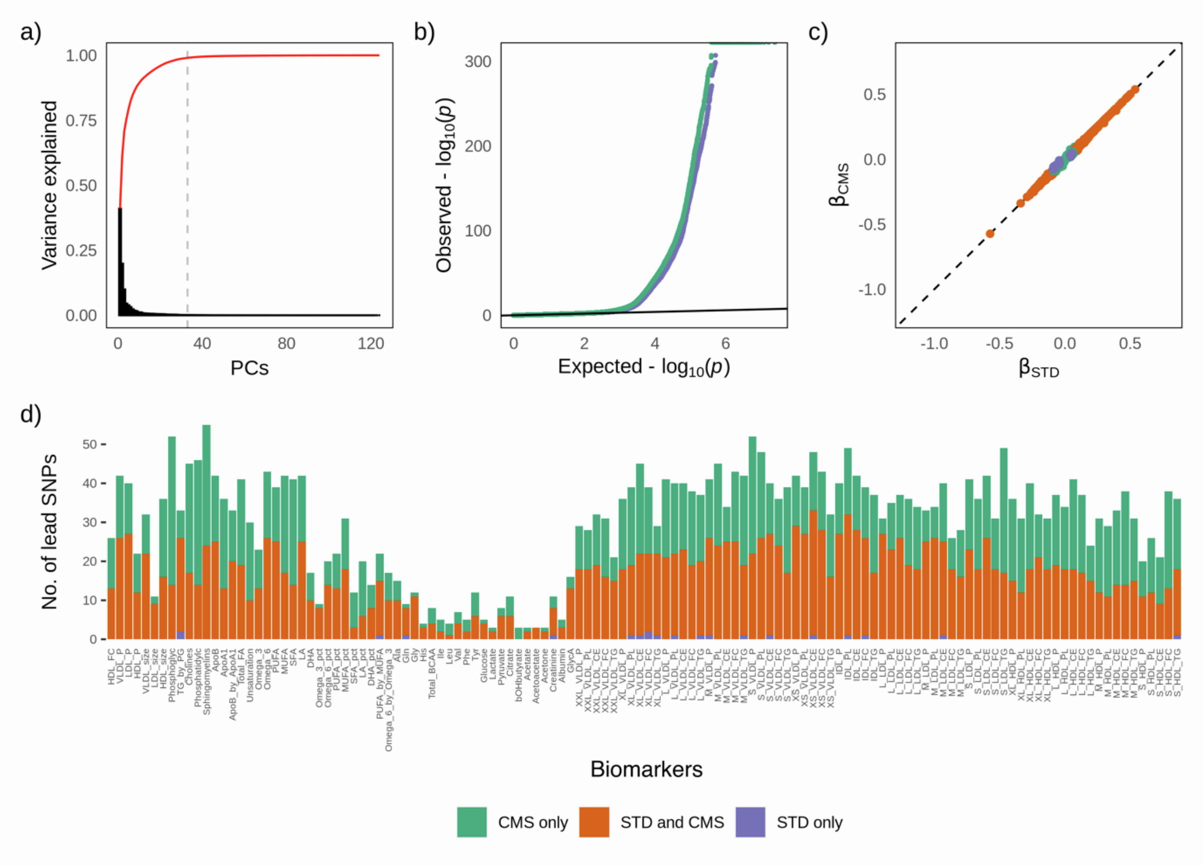

Supplementary Fig 2. GWAS quality control plots for healthy control cohort.
A) The variance explained for each principal component (PC) is shown, with the red curve indicating the cumulative variance and the grey dashed vertical line representing the 33rd PC (explaining 99% of the biomarker variance). B) QQ plots show the observed p-values for CMS (green) and STD (purple) where λ_{CMS}=0.82 and λ_{STD}= 1.05. C) Regression coefficients for significant SNP-regions estimated by CMS and STD are shown where Pearson's r=0.997. D) Comparison of the number of SNP region-metabolite associations found by CMS and STD. Significant SNPs at P<1.51e-09 determined by CMS only are shown in green, both CMS and STD are shown in orange, and STD only are shown in purple. Related to Figure 1.

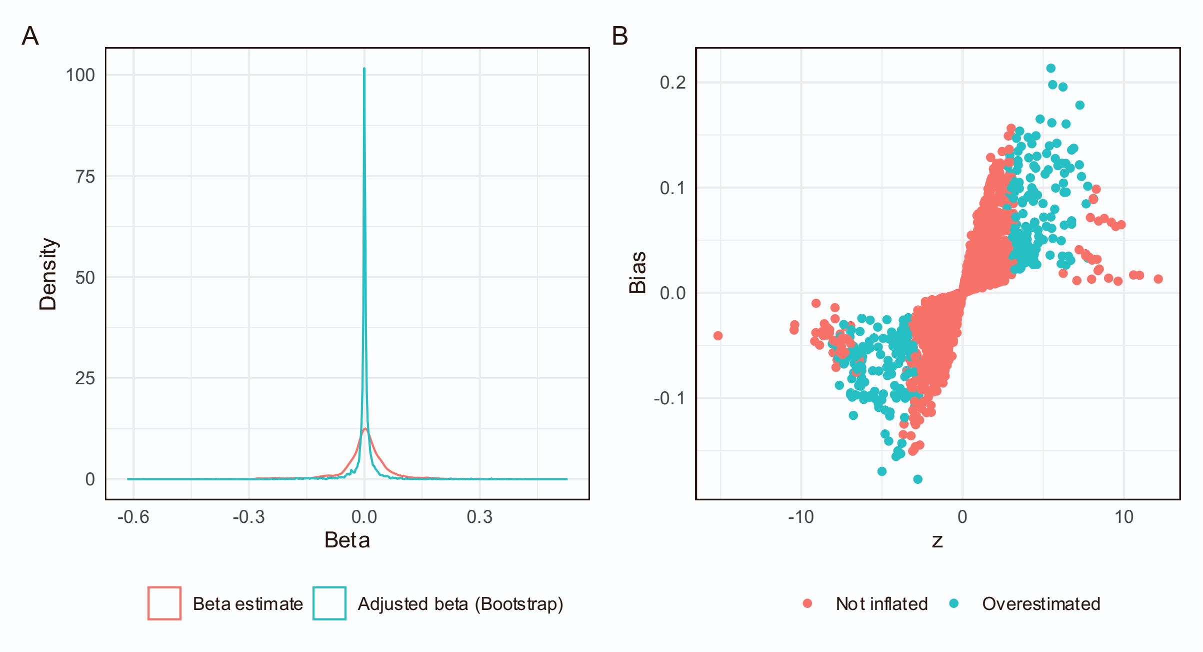

Supplementary Figure 3. Visual comparison of raw and adjusted effect sizes for 4,058 ME/CFS associations.

A) Density plot showing the distribution of raw ME/CFS effect sizes (red) and adjusted effect sizes using Bootstrap method (blue). B) Scatter plot depicting the number of effect sizes that were overestimated where bias=raw beta-adjusted beta. An effect was deemed significantly overestimated if |raw betas|>|adjusted beta|+1.96*standard error (shown in blue). All values for adjusted effect sizes can be found in Supplementary Data 6. Related to Figure 2 and STAR Methods.

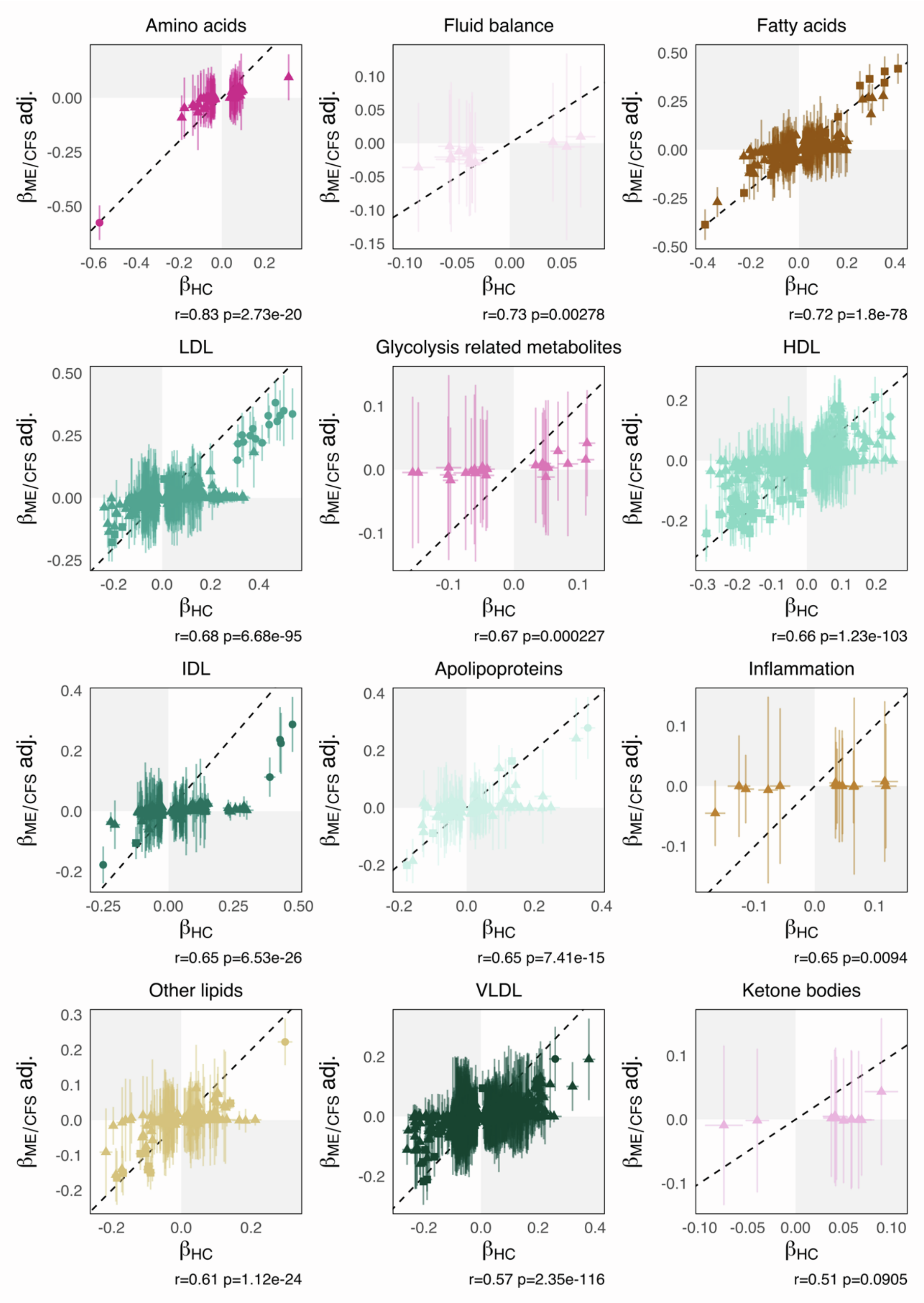

Supplementary Figure 4. Scatter plot of the marginal effect sizes for all the significant associations found in ME/CFS and HC.

Marginal effects are shown for significant associations found in both ME/CFS, which have been corrected for winner's curse using Bootstrap resampling method, and HC (circle), HC only (triangle), and ME/CFS only (square) across 12 biomarker groups. Pearson's correlation coefficient was calculated for each biomarker group, shown in the bottom-left corner of each panel. Panels are organised from highest to lowest correlation. Related to Figure 2.

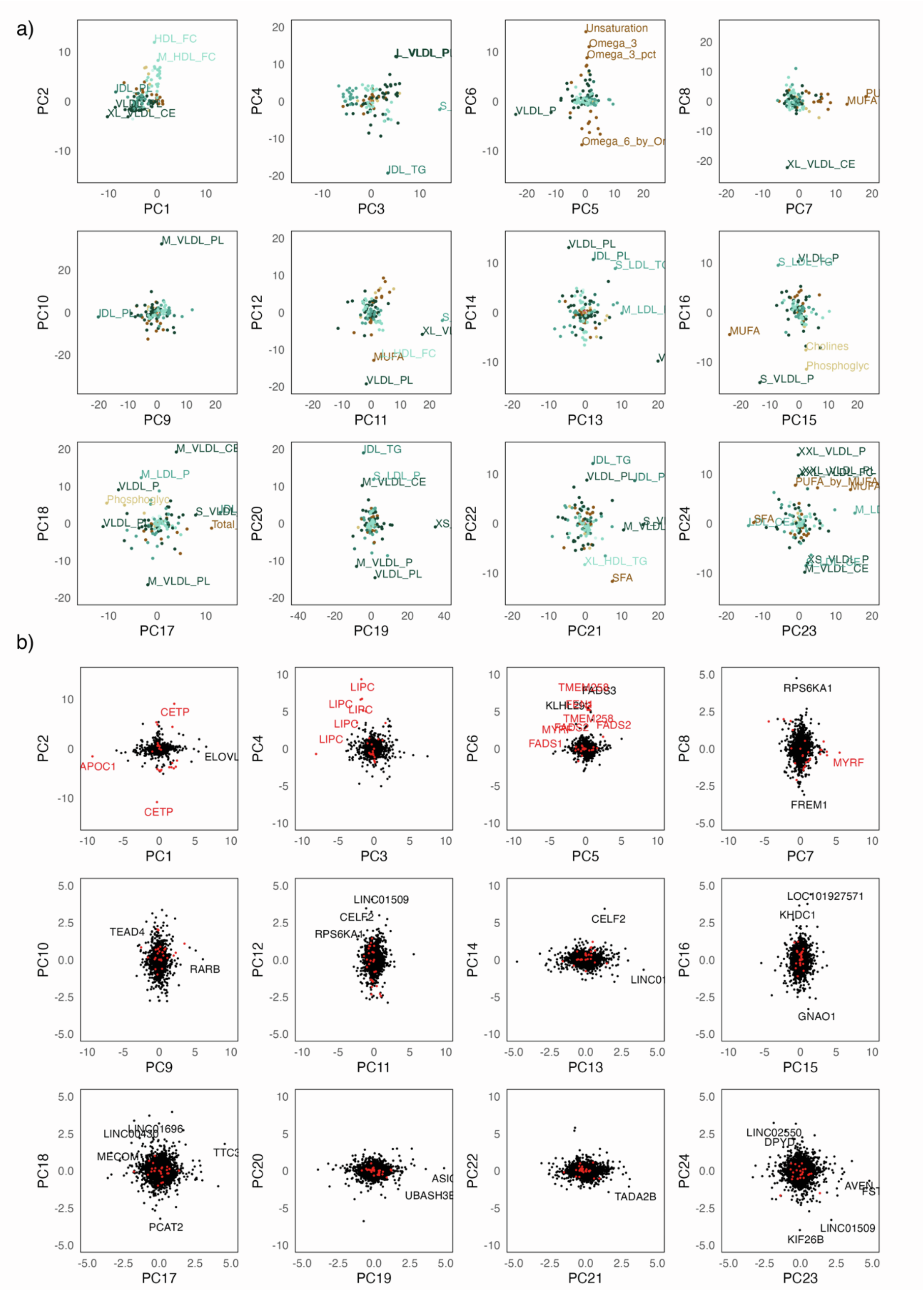

Supplementary Figure 5. Score plots of biomarker and variant projections onto *k=24* latent components using DeGAs in ME/CFS.

DeGAs components corresponding to the latent components derived from A) biomarkers and B) variant features. Features found on the same plane represent a positive association, those facing opposite directions represent an inverse association, and those perpendicular to each other represent no association. Labels in red represent the genes deemed significant from the initial mGWAS summary statistics $p<1.61\times10^{-9}$. Related to Figure 3.

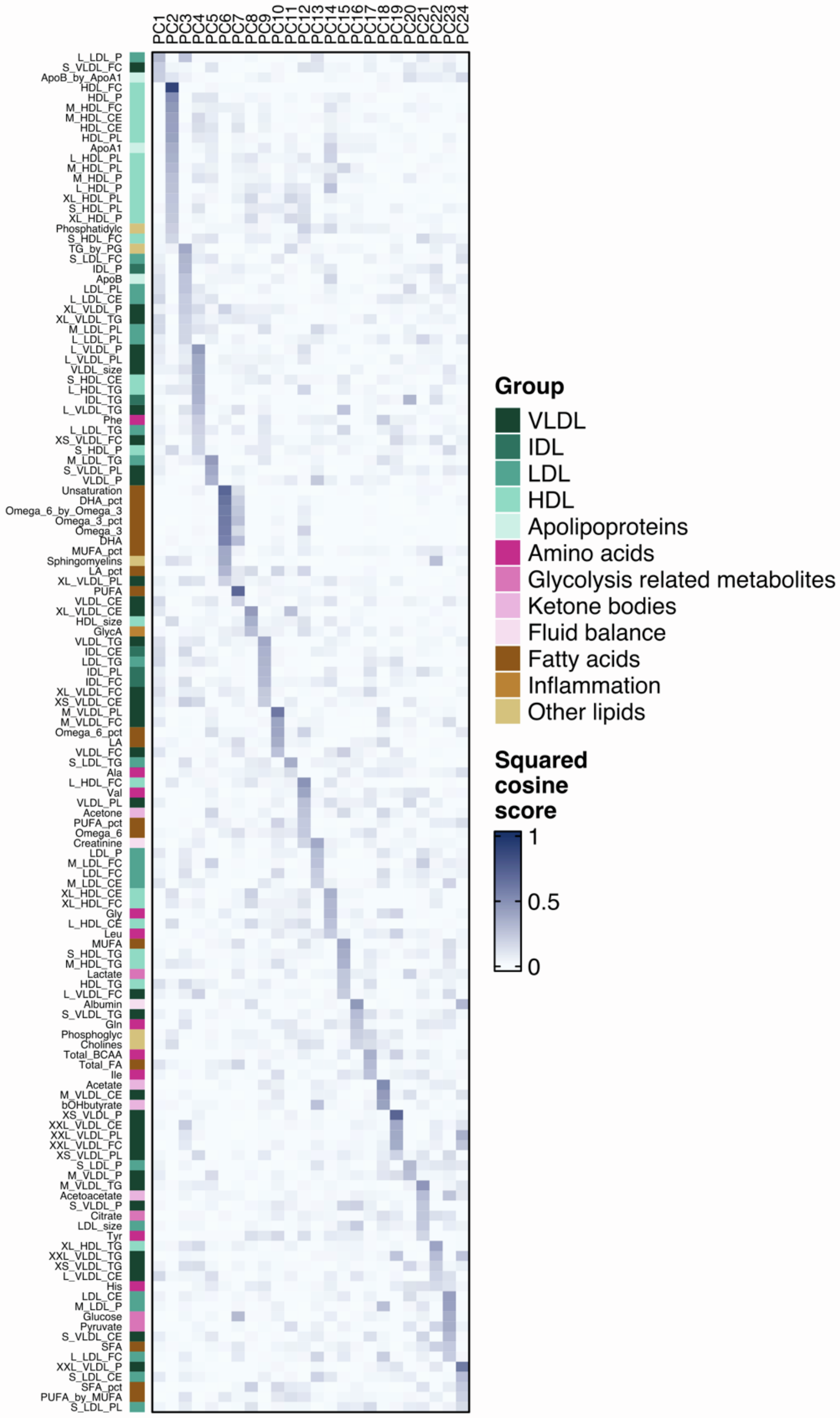

Supplementary Figure 6. Component loadings for biomarkers using square cosine scores in ME/CFS.

Heatmap of the relative importance of each biomarker for a given component derived from DeGAs. The columns show the components (1-24) and the rows show the 135 biomarkers coloured according to their biomarker group (legend). Each cell represents the relative importance based on the square cosine score, ranging from 0-1 shown in the legend. Related to Figure 3.

Supplementary Tables

Supplementary Table 1. Study details of previous ME/CFS UKB case-control GWAS with available summary statistics

**Author**	**Year**	**Cohort**	**Phenotype**	**Case (n)**	**Control (n)**	**Ethnicity**	**Method**	**GWAS Catalogue code**
DecodeME*	2025	DecodeME	ME/CFS case definitions based on CCC, or NAM criteria	15579	259909	European	REGENIE	-
Verma	2024	Veterans Affairs Million Veteran Program	Chronic Fatigue Syndrome (PheCode 798.1)	3891	443093	European	SAIGE	GCST90479178
Pan-UKB	2022	UKB	Chronic Fatigue Syndrome (verbal interview)	1882	418591	European	SAIGE	-
Dönertaş	2021	UKB	Chronic Fatigue Syndrome (verbal interview)	2092	482506	Not reported	BOLT-LMM	GCST90038694
GeneAtlas	2018	UKB	Chronic Fatigue Syndrome (verbal interview)	2017	450247	European	Linear regression	-
Neale lab	2018	UKB	Chronic Fatigue Syndrome (verbal interview)	1659	359482	Not reported	BOLT-LMM	-
Zhou	2018	UKB	Chronic Fatigue Syndrome (PheCode 798.1)	593	405532	British	SAIGE	GCST90436811

UKB: UK Biobank, CCC: Canadian consensus criteria, NAM: National Academy of Medicine
*Details of their GWAS-1 results which included both females and males.
Related to Table 2.

Supplementary Table 2. Biomarker contributions of latent components in ME/CFS.

PCs	Fatty acids	HDL	VLDL	LDL	IDL	Other lipids	Apolipoproteins	Amino acids	Ketone bodies	Inflammation	Fluid balance	Glycolysis related
1	0.0763	0.0335	**0.5313**	0.2785	0.0313	0.0252	0.0239	5.77E-06	8.68E-06	1.41E-05	2.97E-06	4.06E-07
2	0.0458	**0.6748**	0.0639	0.0091	0.0089	0.1392	0.0583	3.92E-06	2.24E-06	1.64E-07	8.31E-07	9.66E-07
3	0.0451	0.0774	0.3104	**0.4578**	0.0667	0.0176	0.0249	1.14E-05	1.13E-05	1.59E-05	7.17E-06	3.48E-06
4	0.0258	0.1657	**0.5886**	0.1269	0.0806	0.0069	0.0055	1.06E-05	9.76E-06	2.71E-05	1.32E-05	1.67E-06
5	0.0964	0.1187	**0.5105**	0.2018	0.0395	0.0211	0.0120	4.39E-05	2.04E-05	7.28E-06	4.51E-08	5.05E-06
6	**0.8253**	0.0170	0.1240	0.0079	0.0137	0.0107	0.0013	1.23E-05	1.06E-05	4.83E-05	1.94E-06	1.64E-06
7	**0.5069**	0.1018	0.2619	0.0530	0.0239	0.0447	0.0077	1.76E-05	7.89E-06	6.78E-05	9.09E-06	1.09E-05
8	0.0554	0.1898	**0.6124**	0.0965	0.0079	0.0328	0.0050	1.84E-05	2.81E-06	5.96E-05	9.41E-07	2.12E-06
9	0.0892	0.0868	**0.3748**	0.3071	0.1104	0.0282	0.0034	1.41E-05	2.63E-05	4.63E-05	1.27E-07	3.27E-06
10	0.2719	0.0599	**0.5778**	0.0577	0.0131	0.0106	0.0088	1.20E-04	7.51E-06	2.83E-05	1.04E-05	7.82E-06
11	0.1357	0.1174	**0.5515**	0.1549	0.0158	0.0190	0.0056	2.12E-05	1.81E-05	1.27E-05	7.27E-07	1.75E-05
12	**0.4003**	0.1735	0.2512	0.0388	0.0046	0.1297	0.0017	7.07E-05	2.15E-05	1.08E-04	3.81E-05	1.55E-06
13	0.0617	0.0296	0.3591	**0.4499**	0.0313	0.0677	0.0005	3.12E-05	1.43E-04	1.98E-05	2.87E-05	7.54E-06
14	0.0501	0.2008	**0.4381**	0.2027	0.0793	0.0089	0.0200	3.60E-05	1.63E-05	1.44E-06	1.09E-05	1.38E-05
15	0.1948	0.2095	**0.4216**	0.1250	0.0231	0.0185	0.0074	1.08E-04	1.52E-05	4.66E-05	3.90E-06	1.05E-05
16	0.1174	0.0516	**0.6109**	0.0750	0.0117	0.1272	0.0062	4.19E-05	1.44E-05	6.56E-07	3.58E-06	1.20E-05
17	0.1388	0.0608	**0.4520**	0.1058	0.0479	0.1927	0.0019	7.70E-05	2.69E-05	5.31E-05	1.30E-05	1.14E-05
18	0.0686	0.0200	**0.4183**	0.3590	0.0640	0.0628	0.0072	6.72E-05	6.60E-05	1.07E-06	1.68E-06	4.14E-06
19	0.0541	0.0307	**0.6647**	0.1375	0.0925	0.0134	0.0068	1.17E-04	1.11E-05	5.35E-05	6.52E-06	3.51E-06
20	0.0557	0.0287	**0.3908**	0.3673	0.1270	0.0041	0.0262	6.07E-05	3.44E-06	8.73E-06	5.75E-06	2.22E-05
21	0.1370	0.0364	**0.3545**	0.2436	0.2032	0.0163	0.0087	1.46E-04	4.21E-05	8.45E-06	7.35E-07	1.65E-05
22	0.2495	0.0625	**0.3541**	0.1281	0.1616	0.0346	0.0096	1.27E-05	6.72E-06	9.75E-06	5.64E-06	1.71E-05
23	0.1481	0.0919	**0.3565**	0.3522	0.0386	0.0021	0.0105	2.64E-05	5.68E-05	2.65E-05	5.13E-06	1.50E-05
24	0.2110	0.0259	**0.4976**	0.1722	0.0584	0.0099	0.0249	4.18E-05	2.98E-05	1.39E-07	5.77E-06	1.31E-05

Contributions are shown as percentages 0-1. Bolded values are the largest contributions for each PC.
Related to Figure 3.
